# Isolation, probiotic properties, and whole-genome analysis of *P. acidilactici* M22 from feline milk: a promising candidate for simulated pet milk formulations

**DOI:** 10.3389/fmicb.2025.1664636

**Published:** 2025-08-29

**Authors:** Xinyu Gong, Xue Wang, Lu Chen, Zhengping Wang, Jun Han, Min Wen

**Affiliations:** ^1^Shandong Key Laboratory of Applied Technology for Protein and Peptide Drugs, Institute of Biopharmaceutical Research, Liaocheng University, Liaocheng, China; ^2^Pet Nutrition Research and Development Center, Gambol Pet Group Co., Ltd., Liaocheng, Shandong, China

**Keywords:** Ragdoll cat, feline milk, complete genome sequence, probiotic properties, *P. acidilactici*

## Abstract

Breast milk constitutes a rich source of bacteria essential for the establishment and modulation of neonatal microbiota, playing a critical role in neonatal health. However, probiotic strains are rarely identified in feline milk. This study successfully isolated *Pediococcus acidilactici* M22 from feline milk and comprehensively evaluated its potential as a probiotic candidate for simulated pet milk formulations. Strain M22 demonstrated high tolerance to acidic conditions (survival rates of 59.93%, 94.15%, and 95.06% at pH 2.5, 3.0, and 4.0, respectively) and bile salts (survival rates of 96.01%, 96.35%, and 84.38% at 0.1%, 0.2%, and 0.3% concentrations, respectively). Furthermore, M22 exhibited strong adhesion (69.23%) to Caco-2 intestinal epithelial cells, Strong suppression of *Escherichia coli O157*, *Staphylococcus aureus*, and *Salmonella Typhimurium SL1344*, a negative hemolytic phenotype, and sensitivity to antibiotics (e.g., gentamicin). Conversely, tests on *C57BL/6* mice, which were randomly split into four groups, showed that giving M22 orally did not harm the mice, raised serum SOD levels, and lowered MDA and BUN levels. These findings indicate that oral administration of M22 significantly enhances the *in vivo* antioxidant capacity of mice. Further more, genomic analysis revealed 1,960 coding sequences, two CRISPR loci, five genomic islands, and two prophage regions. Collectively, these findings indicate that *P. acidilactici* M22 possesses significant potential as a probiotic to improve the health and well-being of pets.

## 1 Introduction

For millennia, cats and dogs have coexisted with humans as cherished companion animals (Grześkowiak et al., 2015). Concurrent with rising demand for companionship, pet owners exhibit increasing interest in health management strategies and nutritional interventions aimed at enhancing animal well-being and longevity (Deng and Swanson, 2015). The gut microbiome is essential for sustaining the health of the host through various mechanisms, including facilitation of nutrient digestion and absorption, preservation of intestinal barrier integrity, modulation of host immunity, and inhibition of pathogenic bacterial adhesion and activity (Yang and Wu, 2023). For the control of pathogenic bacteria, antibiotics are commonly utilized for disease treatment and prevention in companion animals (Dias et al., 2024). However, excessive and inappropriate antibiotic use contributes to escalating issues of intestinal microbiota dysbiosis and antibiotic resistance (Marco-Fuertes et al., 2022). Furthermore, frequent and close contact between pets and their owners raises the chances of bacterial resistance being transferred, posing significant health risks to both animals and humans (Shao et al., 2021). Therefore, developing safe and effective antibiotic alternatives represents a critical research priority (Wan et al., 2019).

In recent years, probiotics are being used more frequently as a substitute for antibiotics in preventing and treating bacterial infections in pet health care (Marco-Fuertes et al., 2022). Lactic acid bacteria (LAB), which are prevalent in nature and typically present in fermented foods, as well as in the digestive systems and milk of both humans and animals (Gopalakrishna and Hand, 2020), have garnered significant attention. Among LAB, *P. acidilactici*, known for its substantial industrial and biomedical application potential, has gained considerable attention in recent years (Sanders et al., 2019). Previous studies have demonstrated that *P. acidilactici* confers probiotic benefits through various mechanisms, including the regulation of gut microbiota composition (La Fata et al., 2018), enhancement of host immune responses (Riaz Rajoka et al., 2017), maintenance of intestinal barrier integrity, and inhibition of pathogen growth and colonization (Han et al., 2017; Kim and Kang, 2019). Current research indicates that host-derived probiotic strains generally exhibit superior adaptability and stability in terms of colonization efficiency, immunomodulation, and infection prevention compared to non-host-derived strains (Wang et al., 2025).

Breast milk is rich in diverse bacteria, an increasing amount of research indicates that microbes from milk are crucial in forming the initial gut microbiota in infants and are vital for developing neonatal immunity (Gopalakrishna and Hand, 2020). Studies based on molecular biology and microbiological techniques have demonstrated that the milk microbiota of mammals is highly diverse and complex, with dominant genera including *Staphylococcus* (in dogs), *Streptococcus* (in humans), *Serratia* (in cattle), and *Corynebacterium* (in goat) (Ge et al., 2021). These microorganisms might help prevent harmful invasions and aid in the development of the immune system when it is still immature. Unfortunately, breastfeeding is not always feasible in real-life situations, highlighting the need to develop innovative formula alternatives that closely replicate the biological functions of breast milk. The microbiota in breast milk comes from safe sources and shows a high degree of diversity. Indeed, studies conducted previously have confirmed LAB’s presence in breast milk and their beneficial roles in establishing the early gut microbiota, promoting immune development, and protecting against pathogenic colonization. However, research on LAB derived from feline milk remains limited, particularly with respect to systematic studies involving strain isolation, identification, and functional probiotic characterization (Zimmermann and Curtis, 2020).

During this study, a *P. acidilactici* strain, referred to as M22, was extracted from the milk of a healthy feline. Extensive *in vitro* and *in vivo* evaluations were performed to assess its probiotic characteristics. Moreover, whole-genome sequencing and functional annotation using high-throughput sequencing technology were performed to explore the potential probiotic mechanisms and safety profile of M22 at the genomic level. This work not only enriches the microbial resource bank of feline milk-derived lactic acid bacteria but also provides theoretical support for the development and functional application of probiotics for pets.

## 2 Materials and methods

### 2.1 Collection and preparation of samples

Two healthy Ragdoll cats were randomly selected as donors. All animals had not received any antibiotic or probiotic treatment within the past 90 days (Zhao et al., 2025). Before sample collection, the nipples and surrounding skin of the lactating cats were thoroughly disinfected using 75% ethanol swabs. The first 2–3 drops of milk were discarded to minimize contamination, and the subsequent milk was collected into sterile centrifuge tubes. The collected milk samples were then diluted 1:9 (v/v) with sterile PBS, and immediately transported to the laboratory under refrigerated conditions.

### 2.2 Isolation and characterization

Milk samples were collected under sterile conditions and serially diluted in sterile saline for subsequent analysis. A 100 μL aliquot from each dilution was spread evenly onto MRS agar plates (Haibo, China) and grown at 37 °C for 48 h. After three consecutive subcultures, candidate probiotic strains were preserved in 30% (w/v) glycerol (Sigma-Aldrich, USA) at −80 °C. Shanghai Majorbio Bio-pharm Technology sequenced the amplified products (Shanghai, China). Phylogenetic analysis and tree construction were carried out using MEGA version 7.0 (Kumar et al., 2016).

During the isolation and screening process, a total of 11 probiotic strains were obtained, including 6 strains of *P. acidilactici*, 3 strains of *Pediococcus pentosaceus*, and 2 strains of *Enterococcus faecium*. Based on the preliminary acid tolerance test at pH 2.5, strain M22 exhibited superior resistance and was selected for subsequent experiments. Bacterial suspensions were cultured in MRS broth at 37 °C until reaching the logarithmic growth phase. Cell density was adjusted to the desired concentration using optical density measurements at 600 nm (OD600) and a previously established OD–CFU calibration curve. The suspensions were normalized to 1 × 109 CFU/mL prior to further experiments. In this study, we employed *P. acidilactici* strain M22, which had been previously isolated and taxonomically identified in our laboratory and is registered at the China Center for Type Culture Collection under accession number CCTCC NO. 31001.

### 2.3 Antimicrobial ability

Cultivation of M22 was carried out in MRS broth (Haibo, China) at 37 °C for 24 h, The culture was then spun at 8,000 rpm for 5 min to separate the cell-free supernatant. The bacterial was washed twice with PBS (0.1 mol/L) and resuspended to a concentration of 1 × 10^9^ CFU/mL to prepare the bacterial suspension. *Escherichia coli* O157, *Salmonella Typhimurium SL1344*, and *Staphylococcus aureus*, were cultured in LB broth (Haibo, China) at 37 °C for 20 h and brought to 1 × 10^7^ CFU/mL for subsequent use. Punch wells on LB broth (Haibo, China) using Oxford cups and 200 μL of bacterial suspension, cell-free supernatant, bacterial precipitate, and each well was supplemented with PBS. Following 24 h of incubation at 37 °C, the diameter of the inhibition zone was measured.

### 2.4 Characterization of probiotic

#### 2.4.1 Acid output curves and growth

Overnight cultures were inoculated into MRS broth at a 2% (v/v) ratio, with sterile MRS broth used as the blank control. During anaerobic incubation at 37 °C, samples were collected every 2 h to measure the OD_600_ and pH.

#### 2.4.2 Acid and bile salt resistance

The procedure was conducted based on the method reported by Safi et al. (2023), with minor modifications. To evaluate acid tolerance, MRS broth was acidified to pH 2.5, 3.0, and 4.0 using 1 mol/L HCl, sterilized, and rechecked after sterilization. 100 ul of overnight cultures were added to 4.9 mL of MRS broth at different pH levels and cultured under anaerobic conditions at 37 °C. Samples were collected at 0 h and 3 h, serially diluted, and plated for cultivation. The plates were incubated anaerobically at 37 °C for 24 h to determine the number of viable cells. The initial cell count at 0 h was designated as N_0_, and the cell count at 3 h as N. Calculated using Equation 1.


3hSurvivalrate(%)=log(N)/log(N)0×100%

For bile salt tolerance testing, MRS broth was prepared containing bile salts at final concentrations of 0.1%, 0.2%, and 0.3% (w/v). A volume of 100 μL from overnight cultures were added to 4.9 mL of MRS broth either including bile salts or bile salt-free. Samples were collected at 0 and 3 h and processed similarly to the acid tolerance assay.

### 2.5 Adhesion to human colon carcinoma (Caco-2) cells

High-glucose DMEM supplemented with 20% fetal bovine serum and antibiotics was used to revive Caco-2 cells (Beijing Solarbio Science & Technology Co., Ltd., China) at 37 °C in a humidified 5% CO_2_ incubator. Culture medium was renewed every 48 h. Upon reaching 70%–80% confluence, Cells were harvested and subcultured after treatment with 0.25% trypsin-EDTA.

#### 2.5.1 FITC labeling of bacteria

Bacterial labeling was performed based on the method of Kaktcham et al. (2018), with necessary modifications. To prepare a 500 μg/mL FITC solution, 0.5 g of FITC was dissolved in 1 mL of DMSO, followed by a 1000-fold dilution. Cells were pelleted by centrifugation (4 °C, 4000 rpm, 5 min), washed repeatedly three times using sterile PBS and resuspended in 1 mL FITC working solution. Following incubation in the dark at 37 °C for 2 h, the labeled bacteria were centrifuged, washed repeatedly three times using sterile PBS to remove excess FITC, and resuspended in DMEM.

#### 2.5.2 Adhesion assay

A suspension of Caco-2 cells at 5 × 10^5^ cells/mL was seeded into 24-well plates (1 mL per well) and cultured for 24 h to form a confluent monolayer. Following PBS washing, 600 μL of FITC-labeled bacterial suspension was added and incubated anaerobically at 37 °C for 2 h. Wells were then washed three times with PBS to remove non-adherent bacteria. Then, 300 μL of 0.25% trypsin solution was added to detach the cells and halt digestion with DMEM. Subsequently, fluorescence intensity was measured at an excitation wavelength of 485 nm and an emission wavelength of 530 nm. Adhesion rate (%) was calculated as: Adhesion rate (%) = (R/R_0_) × 100%

where R_0_ is the initial fluorescence intensity before adhesion, and R is the fluorescence intensity after adhesion.

### 2.6 Antioxidant activity assay

Prior to the experiment, the culture was standardized to 1 × 10^9^ CFU/mL. The DPPH radical scavenging activity was evaluated using a commercial DPPH assay kit (Nanjing Jiancheng Bioengineering Institute, China), The ability to scavenge O2- was assessed using the Superoxide Anion Scavenging Capacity Kit (Solarbio, China), following the manufacturer’s instructions. The solution was prepared by combining 7 mM ABTS with 2.45 mM potassium persulfate and incubating it in the dark for 12 h. Mix 20 μL of the sample with 200 μL of the working solution thoroughly. Incubate the mixture for 20 min at room temperature protected from light, then measure the absorbance at 734 nm.

### 2.7 Evaluation of the safety profile of isolated strains

#### 2.7.1 Hemolytic activity

Hemolytic activity was evaluated by streaking a small amount of bacterial culture onto Columbia blood agar plates, then subjected to anaerobic incubation at 37 °C for 24–36 h. Hemolysis was assessed based on the appearance of hemolytic zones: α-hemolysis (greenish discoloration), β-hemolysis (clear, complete lysis), and γ-hemolysis (no hemolysis) (Nataraj et al., 2023).

#### 2.7.2 Antibiotic susceptibility

Prior to the experiment, the culture was standardized to 1 × 10^9^ CFU/mL. Next, 100 μL of the bacterial suspension was inoculated onto MRS agar plates prepared in advance. Antibiotic disks (5–6 per plate) were placed evenly on the surface. After pre-diffusion at 4 °C for 12 h, the plates were grown anaerobically at 37 °C for 12 h. Antibiotic susceptibility was determined through measurement of the diameter of the inhibition zones in accordance with the disk manufacturer’s guidelines (Nataraj et al., 2023).

### 2.8 *In vivo* safety assessment of the M22 in mice

#### 2.8.1 Animal experiments

Forty ICR mice (provided by Nanjing Huimiaoxin, China) randomly divided into four groups following a 7-days acclimation period under controlled conditions (25 °C, unlimited access to food and water). The control group (C) received 200 ul of sterile saline daily by gavage feeding for 28 days, while the low-dose (L), medium-dose (M), and high-dose (H) groups were administered 0.2 mL of *P. acidilactici* M22 suspensions at concentrations of 1 × 10^7^, 1 × 10^8^, and 1 × 10^9^ CFU/mL, respectively. Throughout the experimental period, daily observations of clinical signs, including diarrhea, were conducted in the mice, with body weights measured weekly. At the conclusion of the trial, mice underwent a 12 h fasting period. After collection from the orbital sinus, blood samples were centrifuged at 3000 rpm for 15 min at 4 °C, serum was isolated for subsequent biochemical testing. Following euthanasia, mice were dissected, viscera examined, and organs were excised and their weights recorded. The organ coefficient was determined by calculating (organ weight/body weight) × 100% (Li et al., 2019).

#### 2.8.2 Serum biochemical parameters in mice

Commercial assay kits were used to measure biochemical markers in serum, such as liver and kidney function markers and antioxidant indices (Jiancheng Bioengineering Institute, China). Measurements were performed with an automated biochemical analyzer (IDEXX, America) or enzyme-linked immunosorbent assay (ELISA) kits according to the manufacturers’ protocols.

### 2.9 Whole-genome sequencing

#### 2.9.1 Genomic DNA extraction

The M22 was streaked on MRS solid medium and cultured at 37 °C for 24 h. Next, a single colony on the solid medium was inoculated into 200 mL of MRS liquid medium and cultured at 37 °C for approximately 16 h at 200 rpm. The cell biomass was harvested after 5 min centrifugation at 8000 × *g*. Genomic DNA of M22 was extracted using Bacterial DNA extraction kit (magnetic beads) (Majorbio, shanghai, China) according to manufacture’s protocol. Purified genomic DNA was quantified and high quality DNA was used to do further research.

#### 2.9.2 Library construction and genome sequencing

Genomic DNA sequencing was performed using a combination of the PacBio Sequel IIe and Illumina sequencing platforms. For Illumina sequencing, genomic DNA from each strain was used to construct sequencing libraries. DNA samples were sheared into 400–500 bp fragments using a Covaris M220 Focused Acoustic Shearer according to the manufacturer’s protocol. Illumina libraries were prepared from these sheared fragments using the NEXTFLEX Rapid DNA-Seq Kit. Briefly, the 5′ ends were first end-repaired and phosphorylated, followed by A-tailing of the 3′ ends and ligation of sequencing adapters. Subsequently, adapter-ligated fragments were enriched by PCR amplification. The resulting libraries were then subjected to paired-end sequencing (2 × 150 bp) on an Illumina NovaSeq 6000 platform (Illumina Inc., San Diego, CA, USA).

For PacBio sequencing, genomic DNA was fragmented to approximately 10 kb in size. The DNA fragments were purified, end-repaired, and ligated with SMRTbell sequencing adapters following the manufacturer’s instructions (Pacific Biosciences, CA). The PacBio library was then prepared and sequenced on a single SMRT cell using standard protocols.

#### 2.9.3 Genome assembly and annotation

The data generated from PacBio Sequel IIe and Illumina platform were used for bioinformatics analysis. All of the analyses were performed using the online platform of Majorbio Cloud Platform^1^ from Shanghai Majorbio Bio-pharm Technology Co., Ltd. The detailed procedures are as follows. The raw Illumina sequencing reads generated from the paired-end library were subjected to quality-filtered using fastp v0.23.0. The HiFi reads generated from the PacBio platform for analysis. Then the clean short reads and HiFi reads were assembled to construct complete genomes using Unicycle v0.4.8 (Wick et al., 2017) and uses Pilon v1.22 to polish the assembly using short-read alignments, reducing the rate of small errors. The final assembled genome was submitted to the NCBI database (accession number SUB15380153). The coding sequences (CDs) of chromosome and plasmid were predicted using Glimmer or Prodigal v2.6.3 (Hyatt et al., 2010) and GeneMarkS (Besemer and Borodovsky, 2005), respectively. tRNA-scan-SE (v 2.0) (Chan and Lowe, 2019) was used for tRNA prediction and Barrnap v0.9^2^ was used for rRNA prediction. The predicted CDs were annotated from NR, Swiss-Prot, Pfam, GO, COG, KEGG and CAZY database using sequence alignment tools such as BLAST, Diamond and HMMER. Briefly, each set of query proteins were aligned with the databases, and annotations of best-matched subjects (*e*-value < 10^–5^) were obtained for gene annotation. Biosynthetic gene clusters (BGCs) of secondary metabolites were identified by antiSMASH v5.1.2 software.

### 2.10 Statistical analysis

All data are expressed as mean ± standard deviation (SD). Statistical analyses were performed using GraphPad Prism version 9.0 (GraphPad Software, San Diego, CA, USA). Comparisons between two groups were conducted using unpaired Student’s *t*-tests, while comparisons among multiple groups were performed using one-way analysis of variance (ANOVA) followed by Tukey’s *post-hoc* test. A *p*-value of < 0.05 was considered statistically significant.

## 3 Results

### 3.1 Identification of M22

**FIGURE 1:**
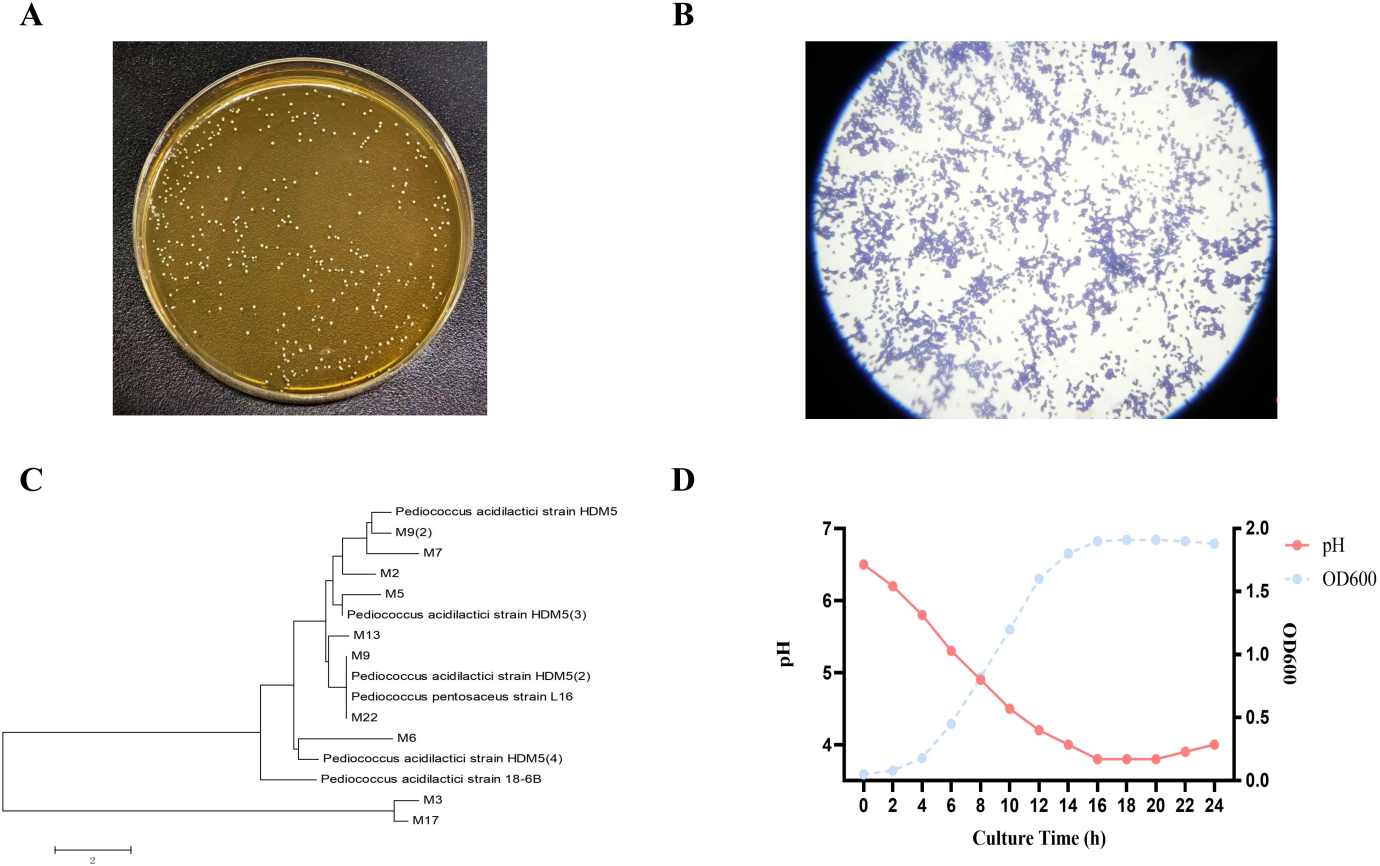
Morphology, staining, phylogenetic analysis, and growth characteristics of strain M22. **(A)** Colony morphology of strain M22 on MRS agar. **(B)** Gram staining results of strain M22. **(C)** Neighbor-joining phylogenetic tree of strain M22 based on 16S rRNA gene sequences. **(D)** Growth curve and pH changes of strain M22 in MRS broth over a 24-h incubation period.

A dominant bacterial strain, designated M22, was isolated from the milk of a healthy lactating cat. On MRS agar, M22 formed round, milky-white colonies with smooth and regular edges (Figure 1A). Gram staining revealed that the cells were Gram-positive cocci, typically observed as single cells or in pairs (Figure 1B). M22’s 16S rRNA gene was sequenced and aligned with reference sequences obtained from the GenBank database. The results showed a 99.81% sequence similarity with *P. acidilactici*, indicating that M22 belongs to this species (Figure 1C).

### 3.2 Antimicrobial ability

**FIGURE 2:**
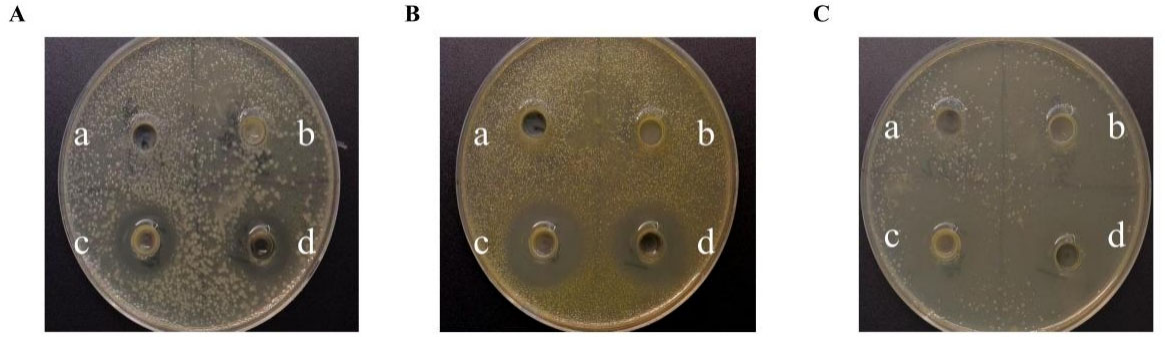
The inhibitory effects of *P. acidilactici* M22 against pathogenic bacteria. **(A)** *Escherichia coli O157*; **(B)** *Staphylococcus aureus*; **(C)** *Salmonella Typhimurium SL1344*. On each LB plate, the following treatments were applied: a, PBS (blank control); b, bacterial pellet of M22; c, bacterial suspension of M22; d, cell-free supernatant of M22.

Figure 2 illustrates the inhibitory activity of strain M22 against pathogenic indicator bacteria. Neither the bacterial protein nor the PBS (blank control) exhibited any inhibitory effect against the pathogenic bacteria (Figure 2a, b). No significant difference was observed between the inhibition zones produced by the bacterial suspension and its supernatant when tested against the same pathogenic strain (Figure 2c, d). The results indicate that M22 exhibits notable antagonistic effects against *Escherichia coli* O157, *Staphylococcus aureus*, and *Salmonella Typhimurium SL1344*.

### 3.3 Probiotic properties

#### 3.3.1 Growth and acid production curve

The strain exhibited a typical bacterial growth curve, characterized by a lag phase during the initial 0–3 h, which was preceded by an exponential growth phase starting around 3 h. The culture entered the stationary phase at around 16 h. In addition, the strain demonstrated a strong acid-producing capability (Figure 1D).

#### 3.3.2 Acid and bile salt resistance

**FIGURE 3:**
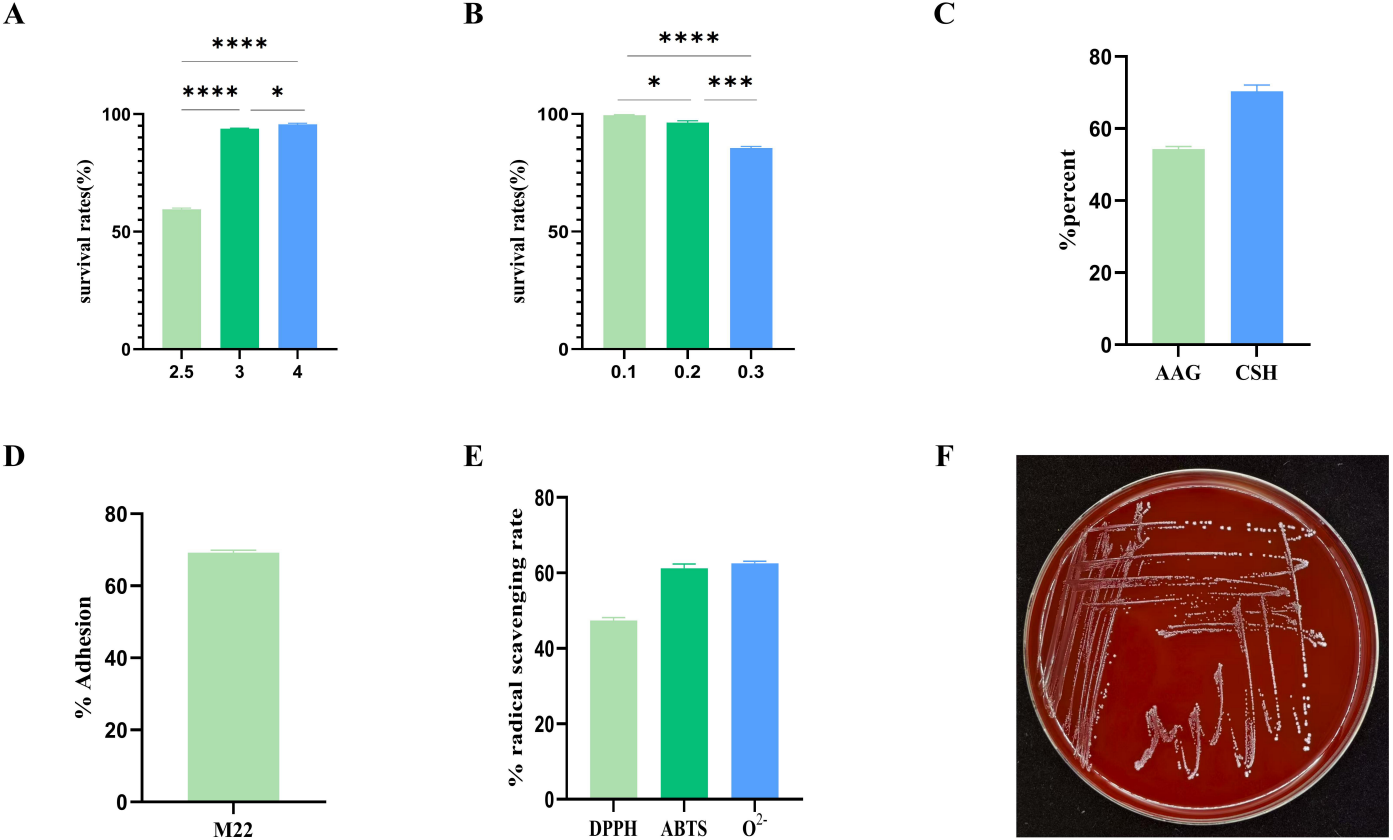
*In vitro* evaluation of probiotic potential. **(A)** pH 2.5–4 acid resistance; **(B)** 0.1%–0.3% bile salt resistance; **(C)** autoaggregation ability and cell surface hydrophobicity; **(D)** Adhesion to Caco-2 cells; **(E)** hemolysis tests; **(F)** radical scavenging rate; ^****^p < 0.001 and *p < 0.01 indicate differences between different types of treatment of M22.

The results indicate that M22 maintained a survival rate of 59.93% at pH 2.5 and 84.38% in the presence of 0.3% bile salts. These findings suggest that M22 possesses strong tolerance to acidic and bile salt conditions, supporting its potential application as an effective probiotic strain (Figures 3A, B).

### 3.4 Adhesion to cells

The strain’s adhesion is also a crucial marker of its probiotic properties *in vitro*. Strain M22 exhibited 55.0% autoaggregation activity and exceeding 65.0% cell surface hydrophobicity (Figure 3C). M22 exhibited a high level of adhesion to Caco-2 cells, with an adhesion rate reaching 69.23% (Figure 3D).

### 3.5 Antioxidant activity

M22 exhibited notable antioxidant activity, with a DPPH radical scavenging rate of 46.15%, an ABTS radical scavenging rate of 63.27%, and a superoxide anion radical scavenging rate of 63.21% (Figure 3E).

### 3.6 Safety assessment of M22

#### 3.6.1 Hemolytic activity

M22 showed no signs of hemolysis (referred to as γ-hemolysis) and is generally considered safe (Figure 3F).

#### 3.6.2 Antimicrobial susceptibility

For safety assessment, M22’s phenotypic antibiotic susceptibility was tested against 12 antibiotics. As shown in the Table 1, M22 exhibited high sensitivity to gentamicin, erythromycin, ampicillin, penicillin, chloramphenicol, and cefazolin (a cephalosporin antibiotic). In contrast, the strain showed resistance to vancomycin, ciprofloxacin, sulfamethoxazole-trimethoprim, dibekacin, and norfloxacin.

**TABLE 1:** Antibiotic resistance of strain M22.

**Antibiotic class**	**Antibiotic**	**Inhibition zone diameter (mm)**	**Susceptibility (S/I/R)**
Aminoglycosides	Gentamicin	20 ± 0.23	S
	Dibekacin	0	R
Glycopeptides	Vancomycin	0	R
Macrolides	Erythromycin	34 ± 0.45	R
β-Lactams	Ampicillin	32 ± 0.15	S
	Penicillin	50 ± 0.22	S
	Cefazolin	36 ± 0.20	S
Tetracyclines	Tetracycline	24 ± 0.05	S
Fluoroquinolones	Norfloxacin	0	R
	Ciprofloxacin	0	R
Sulfonamides/ antimetabolites	Sulfamethoxazole-trimethoprim	0	R
Phenicols	Chloramphenicol	30 ± 0.10	S

### 3.7 Safety evaluation *in vivo*

**FIGURE 4:**
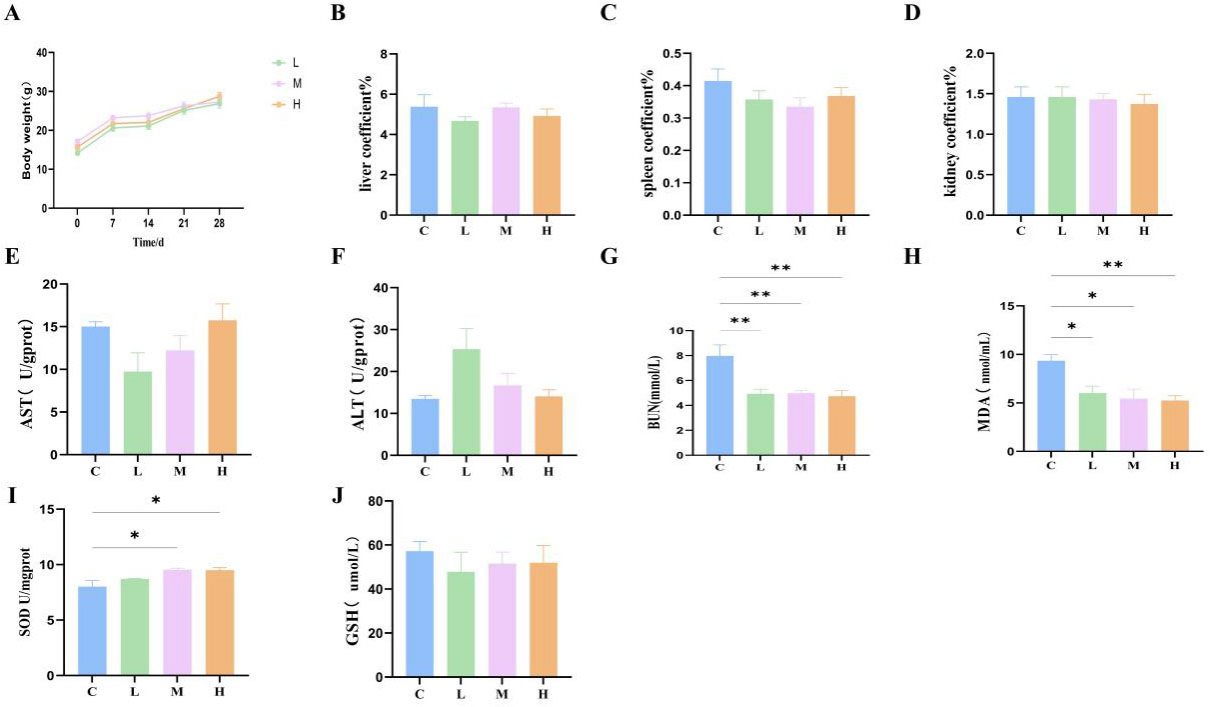
M22 safe evaluation *in vivo*. **(A)** Body weight; **(B)** liver coefficient; **(C)** spleen coefficient; **(D)** kidney coefficient; **(E)** aspartate amino transferase; **(F)** alanine aminotransferase; **(G)** blood urea nitrogen; **(H)** malondialdehyde; **(I)** superoxide dismutase; and **(J)** glutathione in mice. *p < 0.05, ^**^p < 0.01 and indicate differences between different types of treatment of *P. acidilactici* M22, *n* = 6.

The safety of oral administration of M22 at various doses was evaluated in mice. Throughout the entire experimental period, all mice remained active, and no cases of diarrhea, mortality, or other clinical signs of illness were observed. Post-mortem examinations revealed no obvious pathological changes in major organs. No meaningful differences were identified in body weight, liver coefficient, spleen coefficient, or kidney coefficient (Figures 4A–D; *p* > 0.05) between the M22-treated groups and the control group. Moreover, serum levels of AST and ALT in mice administered different doses of M22 exhibited no meaningful differences compared to those in the control group (Figures 4E, F; *p* > 0.05). However, BUN and MDA levels were significantly reduced in the M22-treated groups compared to the control group (Figures 4G, H). In addition, M22 administration notably increased the activity of SOD compared to the control group (Figure 4I), GSH levels did not differ significantly between the groups (Figure 4J).

### 3.8 Complete genome sequencing

**FIGURE 5:**
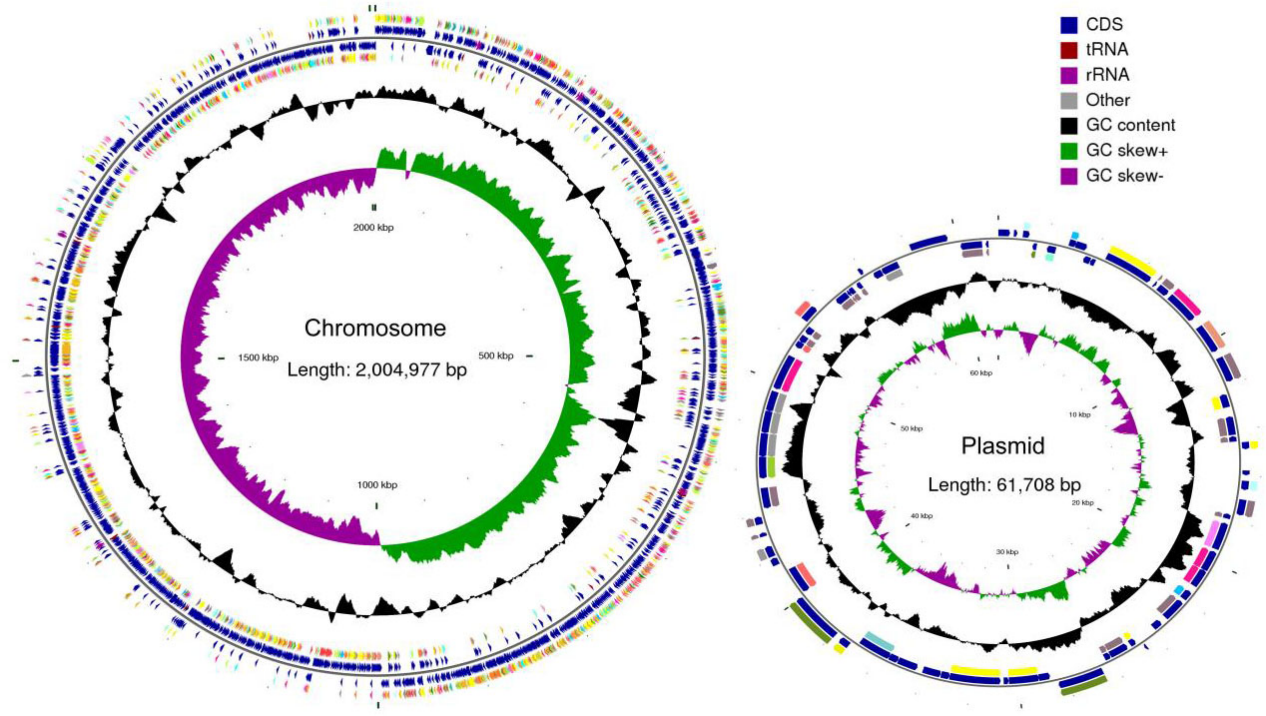
Complete genome map of *P. acidilactici* M22. Circles are indicated from outside to inside: circles 1 and 2 (blue) indicate for-ward and reverse strands, which represent genes for CDS, tRNA and rRNA. Circle 3 (black) indicates the GC percentage of the genome. Circle 4 (purple and green) represents GC skew.

Whole-genome sequencing showed that *P. acidilactici* M22 contains a single chromosome of 2,066,685 bp and one plasmid, with G + C contents of 42.29% and 41.85%, respectively (Figure 5). A total of 1,960 coding sequences (CDSs) were identified, with a cumulative length of 1,778,703 bp. In addition, we identified and annotated 195 pseudogenes, 15 rRNA genes, 59 tRNA genes, and 27 sRNA genes (Table 2).

**TABLE 2:** General genomic information of the strain M22.

**Indicator**	**Number or content**
Chromosome (bp)	2,066,685
Plasmid1 (bp)	61,708
G + C content of chromosome (%)	42.29
G + C content of plasmid1 (%)	41.85
Coding gene numbers	1,960
Total length of coding genes (bp)	1,778,703
Pesudogene size (bp)	195
Pesudogene number	102,765
rRNAs (16S–23S-5S)	15
tRNA	59
sRNA	27

Further analysis identified five genomic islands, two prophage regions, 53 repeat sequences with a total length of 31,605 bp, six insertion sequences, two cytochrome P450 genes, and 173 virulence factors. A total of 66 carbohydrate-active enzymes (CAZymes) were predicted, including 31 glycoside hydrolases (GHs) and 22 glycosyltransferases (GTs) (Table 3).

**TABLE 3:** CAZymes-encoding genes of *Lactobacillus johnsonii* M22.

**CAZymes class definition**	**Gene counts**
Auxiliary activities	5
Carbohydrate esterases	8
Glycoside hydrolases	31
Glycosyl transferases	22

**FIGURE 6:**
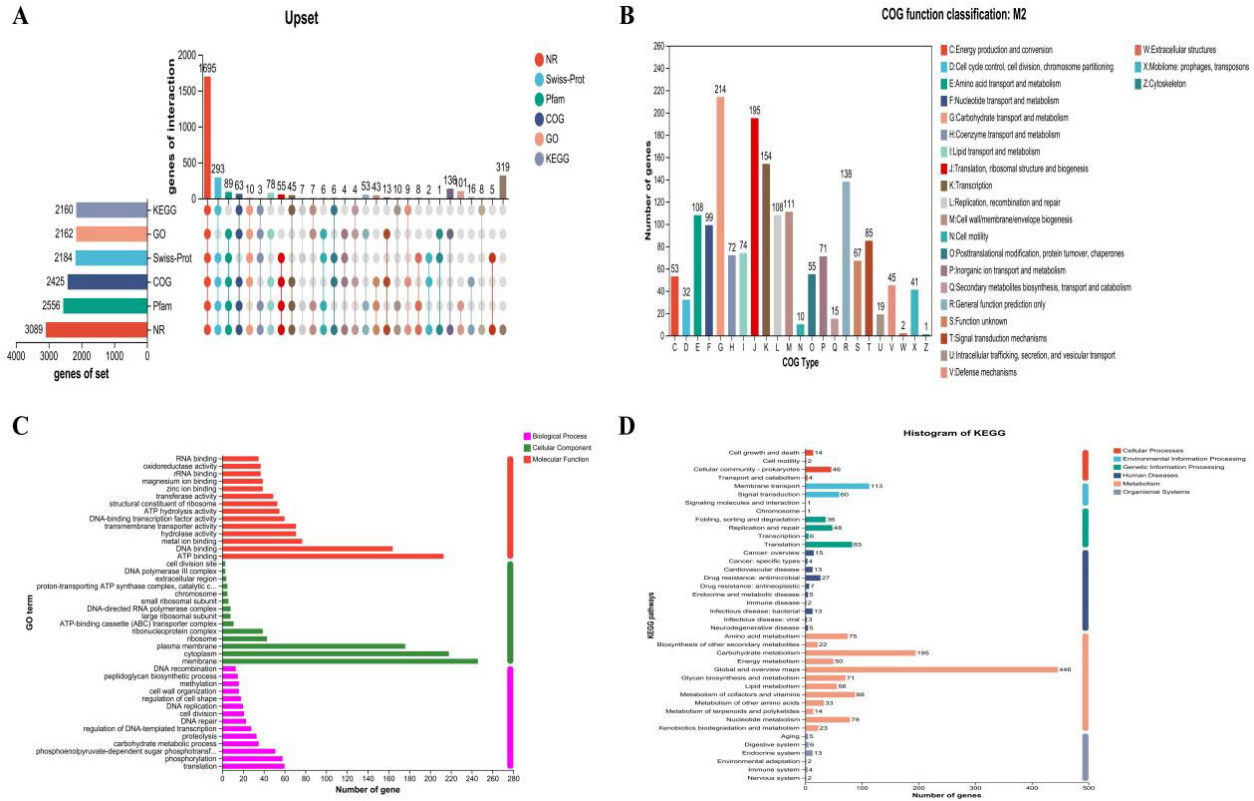
Prediction proteins function of *P. acidilactici* M22. **(A)** General database annotation percentage statistics; **(B)** COG of proteins functional; **(C)** GO analysis and **(D)** KEGG pathways enrichment.

Figures 6A illustrates the genomic map of annotated CDSs in M22 based on general databases. Functional classification using the Clusters of Orthologous Groups (COG) database predicted 1,598 genes (Figure 6B). Then, Gene Ontology (GO) enrichment analysis functional annotation of *P. acidilactici* M22 are shown in Figure 6C. Based on KEGG annotation, 1,484 genes were assigned to 40 functional categories, with major groups including overview maps (284 genes), carbohydrate metabolism (120 genes), and membrane transport (98 genes), as illustrated in Figure 6D.

**FIGURE 7:**
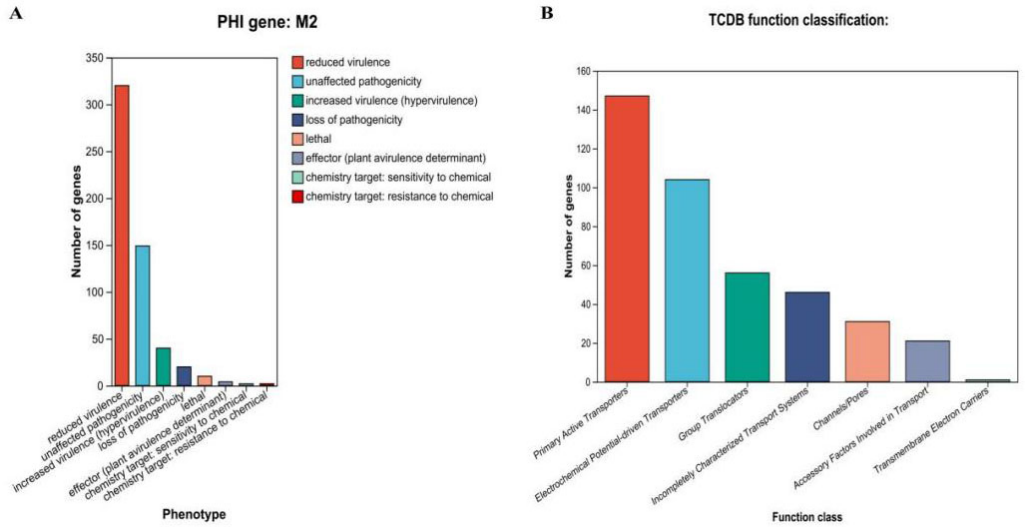
Proprietary database annotations of *P. acidilactici* M22. **(A)** Pathogen host interactions annotations and **(B)** TCDB transporter protein.

We also annotated 475 pathogen-host interaction (PHI) genes (Figure 7A). Additionally, 406 membrane transport proteins were identified, with the largest group being 147 major facilitator superfamily (MFS) transporters (Figure 7B). CRISPR prediction using MinCED (version 0.4.2) revealed that the M22 genome harbors two CRISPR arrays.

## 4 Discussion

Breast milk provides sufficient nutrients to support infant growth and also serves as a critical source of microbial “seeds” that initiate and shape the establishment of the neonate gut microbiota, thereby enhancing the neonate’s tolerance and immune resilience. Therefore, introducing milk-derived microorganisms from a homologous host into simulated pet milk formulations is crucial (Jang et al., 2024). In this study, a strain of *P. acidilactici* (designated M22) was isolated from the milk of a healthy cat. Its probiotic properties and safety were comprehensively evaluated through both *in vitro* and *in vivo* experiments, and its genomic characteristics were analyzed via whole-genome sequencing. The results demonstrated that M22 possesses notable tolerance to gastric acid and bile salts, significant antimicrobial and antioxidant activities, strong adhesion ability to intestinal epithelial cells, and excellent safety, highlighting its potential as a candidate probiotic for simulated pet milk formulation.

In domestic cats, the gastric pH is highly acidic, typically ranging from approximately 1.5 to 3.3 in fasted and postprandial states (Telles et al., 2021). To exert their beneficial effects in the host, probiotics must be able to survive the challenging conditions of the gastrointestinal tract, including exposure to gastric acid and bile salts, in order to survive, adhere to, and Colonize the gut. Therefore, Ability to withstand low pH and elevated bile salt levels is a key factor in screening potential probiotic candidates. Tolerance to acid and bile salts is essential for probiotics to survive the harsh gastrointestinal environment and exert their beneficial effects *in vivo* (Chen et al., 2020). Given that most LAB exhibit limited acid resistance, strain screening under different pH values and bile concentrations is necessary (Kailasapathy and Chin, 2000). M22 showed survival under acidic (pH 2.5) and bile salt (0.3%) conditions, this result is in accordance with previous findings on the gastrointestinal resilience of *P. acidilactici* (Qiao et al., 2021). Highlighting its promise as a candidate probiotic for application in the pet food industry.

Adherence to intestinal epithelial cells is essential for probiotics to successfully colonize and perform their beneficial roles in the host. Caco-2 cells, as a well-established model of intestinal epithelial cells, they are widely used to evaluate the adhesive ability of potential probiotic strains, enabling *in vitro* selection of promising candidates (Schreck and Melzig, 2018). M22 exhibited an adhesion rate of over 69% in Caco-2 cell models, indicating strong colonization potential. High adhesion not only facilitates competitive exclusion of pathogens but also promotes interaction with host cells, contributing to immune modulation (Juntunen et al., 2001). The strong adhesive capacity of M22 further supports its applicability as a host-derived probiotic.

The antimicrobial activity of LAB has been extensively reported (Adetoye et al., 2018). M22 effectively inhibited several common pathogens, including *Escherichia coli* O157, *Staphylococcus aureus*, and *Salmonella Typhimurium SL1344*. Notably, both the culture supernatant and bacterial suspension exhibited inhibitory effects, while bacterial protein extracts did not, suggesting that the antimicrobial activity was attributable to metabolic products (Giles-Gómez et al., 2016). Extensive research has demonstrated that LAB are capable of synthesizing multiple antimicrobial compounds, including short-chain fatty acids (SCFAs), bacteriocins, and hydrogen peroxide, all of which act as potent defense agents against invading pathogens. Among these, organic acids–particularly lactic and acetic acids–can accumulate within the bacterial cytoplasm, leading to a reduction in intracellular pH. This acidification disrupts the metabolic processes of harmful microorganisms, inhibiting their growth and survival (van Zyl et al., 2020). Thus, M22 likely exerts protective effects against intestinal colonization through these natural mechanisms.

Safety is a fundamental requirement for the practical use of probiotics in animals or humans. M22 showed no hemolytic activity (γ-hemolysis) and was sensitive to a range of commonly used antibiotics, complying with international safety assessment criteria for probiotics (Halder et al., 2017). Serum ALT and AST are crucial markers for liver damage diagnosis, and any pathological or toxic injury influences their levels (Thakur et al., 2024). Organ index, a standard parameter in toxicological studies, reflects potential congestion or swelling of affected organs (Michael et al., 2007). *In vivo* evaluation further supported its safety: no adverse clinical signs, abnormal body weight changes, or significant alterations in liver function (ALT, AST) or organ indices were observed following oral administration in mice. BUN is commonly utilized as an important biochemical indicator for assessing kidney function (Melnikov et al., 2002). Beyond its antimicrobial properties, M22 demonstrated promising antioxidant capacity. MDA is a marker of lipid peroxidation and can indirectly reflect the severity of oxidative damage in body tissues. An increase in oxygen levels may cause an overproduction of reactive oxygen species (ROS), which induces oxidative stress, damages cellular proteins and lipids, and can eventually lead to cell death (Amaretti et al., 2013). Probiotic strains with antioxidant properties are therefore beneficial in maintaining host health (Mills et al., 2011). *In vitro* assays confirmed M22’s ability to scavenge free radicals. Animal experiments corroborated these findings: oral administration of M22 significantly increased serum SOD levels, a key enzyme in the first line of antioxidant defense (Zhou et al., 2023). These data suggest that M22 contributes to the host’s antioxidative defense system. M22 significantly reduced serum BUN levels, MDA levels, and elevated superoxide dismutase (SOD) activity, suggesting not only the absence of toxicity but also a positive effect on oxidative stress resistance. These results are consistent with earlier safety studies on *Lactococcus lactis* CIDCA strains (Fan et al., 2023).

Whole-genome sequencing revealed that *P. acidilactici* M22 possesses a circular chromosome of 2,066,685 bp and a plasmid, with G + C contents of 42.29% and 41.85%, respectively. The genome encodes 1,960 coding sequences (CDSs), along with 15 rRNA genes, 59 tRNAs, 27 sRNAs, and 795 pseudogenes, indicating a genetically rich and complex architecture. These genomic features are comparable to previously reported *P. acidilactici* strains isolated from dairy or gastrointestinal environments (Liu et al., 2021; Aziz et al., 2025).

Multiple structural elements were identified, including five genomic islands, two prophage regions, six insertion sequences, and 53 tandem repeats. The presence of two cytochrome P450-encoding genes suggests potential roles in detoxification and oxidative stress adaptation (Nelson, 2013). Genome annotation revealed a total of 66 carbohydrate-active enzymes (CAZymes), including 31 glycoside hydrolases (GHs) and 22 glycosyltransferases (GTs), which are critical for carbohydrate metabolism and survival in the gut (Lombard et al., 2014; Zúñiga et al., 2018).

Kyoto Encyclopedia of Genes and Genomes (KEGG) and COG analyses annotated 1,484 and 1,598 genes respectively, with key functional categories encompassing carbohydrate metabolism, membrane transport, and global regulatory pathways. These pathways contribute to energy acquisition and environmental adaptability, consistent with probiotic strains adapted to the gastrointestinal tract (Zheng et al., 2020).

A total of 173 potential virulence factors were annotated in the VFDB (Virulence Factor Database) for strain M22. Notably, the majority of these genes were annotated in the KEGG and COG databases as being involved in immune modulation and amino acid metabolism. Virulence factors are typically classified into two categories: defensive factors, which protect bacteria from host immunity, and offensive factors, which directly harm the host. However, this classification becomes ambiguous in the context of probiotics, as many defensive genes are indispensable for probiotic survival and function and are increasingly recognized as health-associated factors rather than true virulence determinants. Therefore, the identified virulence-related genes in M22 should not be interpreted as conventional virulence factors, but rather as adaptive elements that enhance the strain’s ability to persist and thrive in ecological niches such as the gastrointestinal tract (Senan et al., 2015). Furthermore, the presence of only two CRISPR loci, as predicted by MinCED, implies moderate phage resistance and genome stability, a desirable trait for industrial probiotic application (Grissa et al., 2007).

Genes associated with host–pathogen interactions (*n* = 475) and membrane transport proteins (*n* = 406, including 147 ABC-type transporters) were identified, Transporter proteins play a crucial role in bacterial life by facilitating nutrient uptake and providing defense against internal and external stresses (Piepenbreier et al., 2017), indicating potential involvement in adhesion, colonization, and nutrient uptake (Feng et al., 2020). Such features enhance probiotic persistence and functionality in the host gastrointestinal tract.

Strains that cause hemolysis can lead to sepsis in the host (van Hoek et al., 2024). Consequently, non-hemolytic bacteria are regarded as vital for superior probiotics. M22 exhibited no hemolytic activity on blood agar plates. Moreover, genomic analysis revealed the absence of non-hemolytic enterotoxin (Nhe) and hemolysin BL (Hbl) genes, which is consistent with the observed lack of hemolytic activity. In conclusion, M22 exhibits excellent probiotic characteristics, including acid and bile tolerance, antimicrobial and antioxidant activities, strong adhesion capacity, and high safety both *in vitro* and *in vivo*. These features support its potential application as a pet probiotic candidate. Future studies using animal models or clinical trials are warranted to further explore its specific roles in modulating gut microbiota and enhancing immunity in companion animals, thereby providing a scientific basis for the development of gut health-promoting probiotic products for pets.

## 5 Conclusion

*P. acidilactici* M22, isolated from feline milk, exhibits strong acid and bile tolerance, antimicrobial and antioxidant activities, and good adhesion to intestinal cells. Genome analysis confirmed its safety and probiotic potential, with no transferable resistance genes. *In vivo* studies showed that M22 enhances antioxidant capacity without toxicity. These findings support M22 as a promising probiotic candidate for improving pet gut health and overall well-being.

## Data Availability

The raw sequence data have been deposited in the NCBI database under accession number PRJNA1274692.

## Footnotes

1
http://cloud.majorbio.com

2
https://github.com/tseemann/barrnap
